# The American Institute, Session of 1882

**Published:** 1882-09

**Authors:** 


### HOMŒOPATHIC PHYSICIAN.

A MONTHLY JOURNAL OF MEDICAL SCIENCE.

"If our school ever gives up the strict inductive method of Hahnemann, we are lost, and deserve only to be mentioned as a caricature, in the history of medicine."—CONSTANTINE HERING.

Vol. II. SEPTEMBER, 1882. No. 9.

### THE AMERICAN INSTITUTE, SESSION OF 1882.

"*Resolved*, That it is the sense of the American Institute that no physician can properly sustain the responsibilities, or fulfill all the duties of his professional relations, unless he enjoys absolute freedom of medical opinion, and unrestricted liberty of professional action, as provided for in the Code of Ethics of this Institute."

In the published journal of this session, this resolution appears as its last act, except that of thanks to the Committee of Arrangements, the newspapers, etc. Who, of all its members, felt that he needed for his support or defense such an utterance from this body? If there were one, it is a fair question—what had this man, or the entire present membership of the Institute, except the honored one who voted "no" on its passage, been doing, that made them conscious of such needed defense? The members of this Institute were supposed to have recognized and accepted the fact of a God-enacted law of healing, when they became members, and that this recognition and acceptance were the foundation of their application for membership, and without these, it is not too much to say, they never could have become members. This is certainly true of applicants in the early years of the history of the Institute. It would seem that, wanting this recognition and acceptance, a man can have no place in this body, this law being the foundation of its organic existence. The Institute was brought into being by a body of very earnest and honest men, who had accepted this law after adequate experience of its truth, and abundant observation of the superior benefits of a practice in obedience to its requirements. Their objective in founding this body, was to extend a knowledge of this law and increase the number of the agents it requires for curing the sick, and to perfect the knowledge of the powers of these agents, that therewith the sick might be the more certainly cured in accordance with the requirements of this law. In this there was no difference of judgment or feeling expressed, in the lengthened discussions of the first session which gave the Institute birth. The original members were of one mind as to the law, its origin, authority and benefits. It was understood then, that applications for membership implied, on the part of the applicant, an obligation to *obey* this law in his clinical duties. If there had been any intimation on the part of an applicant, that when once a member, his practice with the sick was to be a sort of "*a go as you please*" affair, and not at all to be controlled by law, no doubt his application would have been rejected. Why should it not have been then, and why not now? What part can a man have in an organization founded on God's law, who, at the outset, determines to disregard this law whenever the whim seizes him, or whenever he may encounter a difficulty in its administration, greater than his force of will and present knowledge are equal to overcoming? What can such a man do in the work of this Institute, of extending a knowledge of this, God's law, or increasing the confidence of any sane man in it? He shows he has neither knowledge nor confidence himself. This implied pledge to practice in accord with this law—was it the object of this resolution to discharge the present membership of the Institute from their obligations thus supposed to have been assumed? If so, why? Is the law less a law now than when this pledge was implied? Is it less reliable as a guiding factor in the solution of the problem of finding the specific curative for a given sick condition? Are the results of a practice in obedience to this law less beneficial than formerly, or than are those of a practice without other law than the ever varying whim of the prescriber? If the answers to these questions are in the negative, then why this discharge, or why this resolution?

The only obvious reply to this last question, is found in the word so charming to us all—"liberty!" Ours is often called a "*land of liberty*," and so it is. And, therefore, there could be no reasonable call for this resolution. No man had need of it who had been doing his duty loyally. Habitual violators of the law have no right to such defense. Who, of all men on earth, had the least inclination to interfere with the "*liberty*" of any man so doing. But what is "liberty" without law? Socially and civilly such "liberty" is license and anarchy. Professionally, such "liberty" is only anarchy and confusion. "Liberty" *without law* is only satanic and not of God at all. It has never brought benefit to man or to any society of men. To resolve to do as I please in a matter where God has given a law for its governance and guidance, regardless of that law, is in men only a presumption, a folly, a sin. And the resolve of a "liberty" to do this in the matter of obedience or of disobedience to our law, is only and clearly a resolve of liberty to sin grievously against God and man, whenever whim may prompt so to do.*

Am I not at "liberty" to *do* as I please in my professional duties? Certainly not, if you please to violate God's law. You may not be compelled to a responsibility to man for this, but responsibility to law you cannot escape. This, of course, you being a member of the Institute, supposes your acquaintance with the fact of that law, and with its nature and requirements. But "*liberty of opinion*"—shall I not have liberty in this land to entertain such opinions as I choose? Is not this coercion of opinion but other words for tyranny? Liberty to *believe* as one chooses, cannot be denied to any man or body of men. This is his or their right, as far as all other men are concerned. This man may believe *a lie*, if it pleases him, but in so doing, he must accept the consequences of *believing a lie*. He must be supremely stupid if, in this faith he expects to reap the fruits of truth. To be sure, the logic of the resolution, at least of its spirit, declares parity of truth and falsehood. Its spirit only repeats the delusion often given as an excuse for wrong-doing in other matters. "It is no matter

* In treating the sick there is no liberty for any man to do less than the *best possible* for his cure. No amount of "*resolving*" by any body of men, whoever they may be, can create such a liberty. To attempt this by our Institute, was certainly foolish in the highest degree. They were supposed to possess the evidence of the vast superiority of the homœopathic treatment of disease over *all* other treatments, including even the empirical so adroitly recommended, by implication, in this resolution, and to have accepted this evidence as conclusive. With them debate of this question was ended, or was supposed to be, before they became members of this body. They *knew* the superiority of this treatment over all others, as has been shown by recorded statistics of results whenever it has been brought into contrast with other treatments. Being thus, many times and often proved to be best, where is the value of a liberty of these members to practice something else, which, of course, must be worse? These members have no such liberty, nor have they power to create it for themselves. The attempt to do this, or to assert its existence, is only an attempt to be ashamed of. They might, with equal respectability, as well attempt abrogation or substitution of any other of God's laws.

what a man *believes* so long as he is *sincere* in his belief." It is a great matter, and for this reason. No amount of *sincerity* can transform a *lie* into a *truth*. And the results of a man's faith carried into practice will be determined by the character of the objective of that faith. The fruits of truth can never be gathered of lies, however sincere may be the endeavor to gather them. The sum of all then is—freedom of opinion and action in medical matters, as before men—granted. No rightminded man has any desire to tyrannize over his fellow in this matter. But as to the members of the American Institute, and before the law they have professed to accept and obey, the case is different. They cannot escape the responsibility of violation of this law, nor in its practical violations can they ever reap the successes assured to practical obedience. If, then, we put in briefest terms the true character of this resolution, and the action of those who in it declared their "sense" of the proper thing for themselves and all other doctors to do, who will do their duty in accord with this "sense," it will be summed up in—*treason against God and man—against divine law, and all intelligent experience of obedience to this law in prosecution of clinical duty.* In one word the whole character is found, and that word is—*treason.* Treason to Homœopathy, which these members had professed to have accepted as God-given—treason to the Institute, in that it inculcates habitual violations of the law on which it had been founded, whenever the sweet will of these members or other doctors shall choose so to do. Treason against those members of the Institute not present, inasmuch as they are included in this resolve which destroys the homœopathic character of the body, and passes its membership, body and bones, over into eclectics, whether they consent or not. We submit that this treason is neither justifiable nor decent. And more than this, it is treason against the best interests of the sick, which are proved to have been best secured by a practice strictly in accord with the requirements of the homœopathic law. This has been proved by the best homœopathic experience, and by abundant statistics gathered in many lands, from the results of this practice, in many public institutions in those lands, and by a comparison of these with the results of other forms of practice in those lands, and for the same years, and this through long periods of time, the records in each and every instance sustaining the claimed superiority of the homœopathic treatment in accord with law. And yet, this Institute, its only national organized defender, in the face of this record, and before the world, resolves for itself a liberty to violate the demands of this law, and to resort to anything for their cures, though, being members of the Institute, they are supposed to know that the resources for healing, and all the best of them are found and only found in those which have the sanction of this law. Knowing this, they resolve for themselves a liberty to do for their patients something or anything, which, being outside of law, must be less than the best possible, and that duty and responsibility cannot otherwise be fully met. Surely there can be no greater absurdity. There can be no such liberty for these members.

Then, as to the motive of passing such a resolution. We confess we are wholly incapable of imagining any which does not refer its origin infallibly to ignorance and imbecility. "*Can't find the specific remedy the law demands*—then find something else, and we of the Institute will resolve that you are right in thus turning your back on law, and that all other men fail of their duty who will not do the same thing." Did it occur to these resolvers that though *they* could not find the specific remedy there are many who can, and that what one man can do is possible to another, that the record of practice under the law shows immensely better successes than is found in any eclecticism whatever, and that it might have been more to their credit if they had practiced and advocated the needful study required for their deliverance from this fearful imbecility, from which alone this resolution finds its origin and excuse? Hard work, and that alone, and plenty of it, has given to those who possess it the power to find specific remedies for the sick, under the law, and having obtained this power, they have no need of other resorts outside of those required by the law, and their full "*freedom*" under the law is so complete that they have no desire for that other, outside of law, which is in itself only treason to God and man's best interests, and which only ends in confirming imbecility and leaving its seekers in total darkness and anarchy. Whoever heard of Bœnninghausen, Gross, Stapf, or Haynel, or of any of the class of prescribers of whom these may be taken as representatives, calling for a liberty to forsake and transgress the law which dominated all their clinical duties, and obedience to which had given them the successes which had placed them pre-eminent among those who are of the first rank of practical healers? These men had no need of such a liberty, or of the means it is supposed to put into the hands of incompetents, by which they are to find escape from clinical difficulties. They found the law so perfect and universal in its application, and the means of complying with its demands so ample, that nothing more was required by them to attain those successes which have made their names among healers monumental. They found the law so much better as a guide than any tradition, whim or prejudice outside the law, that they never seemed to have wavered in their confidence in it, or to have imagined that any intelligence, knowing their law, its methods and means, could for a moment have resorted to aught else as its superior. These were not the men who discovered Hahnemann's "errors" and "fallacies," or found a chief delight in scolding or disparaging the materia medica he originated. This has been reserved for those who need defense for transgressions of his proclaimed law, and for those other incompetents who have been constrained to acknowledge their inability in materia medica to distinguish the true from the false. These heroes could obey law and use the materia medica successfully, and found both equal to all their needs, and never appeared to be conscious of needing defense or excuse in view of the results of their prescribing, or of a liberty to do something different from a strict compliance with law, knowing that this was sure to bring the best results in healing, and their history justified their faith and their practice. Is there not in the example of these worthies a sharp rebuke of those who, having forsaken law and failed of success, resolve to quiet their consciences (we can see no other objective of this resolution), by resolving that something else is as good, and perhaps better, than law, and tell the world that they, being unable to administer this law, claim the liberty to go after this something else, whatever it may be, as may be decided by any whim, their own or of others, and give this outcry of feebleness to the world as the outcome of the wisdom of the American Institute, it as was found in its session of 1882. We cannot but remember that the worthy dead, who were our associates in organizing this Institute, by their earlier departure have been spared a mortification which their survivors have been made to endure. Nor can we withhold honor from him who had the courage, the knowledge and the conscience to give this resolution his *one* negative vote. His name should be known, and never forgotten, when those worthy of most honor are named.

It may be of interest to inquire what is to be the result of this extremely silly act of this respectable body of physicians? The first result is already accomplished. They have unmistakably stultified themselves. They have repudiated the character given by its founders to the body they represented. The Institute was by these set for the promulgation of a knowledge of that law which is the basis of the science of therapeutics. It was to be the advocate and defender of that science, and that against all antagonists. The founders of the Institute regarded this science, so founded, as exclusive of any pretended accessories or adjuncts based on other laws, as no other law than that of the similars was known which could sustain a science of therapeutics. Nor is there any other known now. And yet these doctors assert liberty to resort to whatever of means, unsupported by any law, and that this is necessary in order "to fufill all the duties of his professional relations," *i. e.*, it is the "sense" of these doctors that anything is just as good as this which the founders of the Institute regarded as an infallible law, and any means are as good, or better, than those which had been proved and made ready to their hands by the labors and sacrifices of those who have given us our materia medica. They virtually say no man can do his whole duty, or discharge all his responsibilities to the sick, who will not cast these aside, and follow whatever of whim may for the time dominate his judgment, or want of it. In this, is it saying too much when we say, when the Institute passed this resolve it stultified itself?

As a second result, already accomplished, when this Institute passed this resolve it ceased, so far as the body so acting had power to produce such a result, to be an institute of *Homœopathy* at all. It abandoned, there and then, all that is characteristic of it, and substituted in the place of its law, the ever-varying whim of individual choice. For law it gave anarchy and confusion, and this with an air which seemed to indicate the members thought they had "*done a good thing!*"

A third result was, disgrace of themselves and all who bear the homœopathic name, so far as this act of the Institute could reach others than those present and voting on this resolution, by its abandonment of the just claim, always heretofore asserted and sustained by all homœopathists, of the superiority of our law to all other systems or methods of healing, and its universality of application to all the needs of professional healers, and of the sick they might be called to care for. And for themselves the deepest possible disgrace, in that while virtually recommending an abandonment of

God's law, at will, they have given no substitute for this to whoever of the simple may find themselves the dupes of this resolved "*freedom.*" To destroy and not construct, this seems to have been their objective, and for this we see no reason to doubt their greater ability.

A fourth result is, they have brought on themselves, and justly, the contempt of their antagonists, for voluntarily and for no adequate cause, having abandoned all which is characteristic of Homœopathy (which as its Institute, they were supposed to exist to promote and defend), without even a challenge from any outside party to make this melancholy surrender. They seem to have been only too eager to do this, just as it might have been if they had found its duties and responsibilities more than they were able or willing to bear. The "*resolve*" will stand before these antagonists and the public as evidences of the same kind of poltroonery which characterizes the act of the suicide, who has not the strength of will or courage to meet the responsibilities of living in the world in which God's Providence had placed him. It stands, and will stand, a feeble imitation of that act of one of our State organizations, by which it was said, and fitly, to have publicly "committed *hari-kari* in the streets." This State organization repented of its folly and repealed its "*resolution,*" being, apparently, ashamed of it. It remains to be seen whether the Institute will find itself possessed of equal grace. P. P. W.

